# The Insights of Microbes’ Roles in Wound Healing: A Comprehensive Review

**DOI:** 10.3390/pharmaceutics13070981

**Published:** 2021-06-29

**Authors:** Thambirajoo Maheswary, Asma Abdullah Nurul, Mh Busra Fauzi

**Affiliations:** 1 Centre for Tissue Engineering and Regenerative Medicine, Faculty of Medicine, Universiti Kebangsaan Malaysia, Cheras, Kuala Lumpur 56000, Malaysia; p110243@siswa.ukm.edu.my; 2 School of Health Sciences, Universiti Sains Malaysia, Kubang Kerian 16150, Malaysia; nurulasma@usm.my

**Keywords:** normal flora, microbes, wound healing, chronic wound, wound infection

## Abstract

A diverse range of normal flora populates the human skin and numbers are relatively different between individuals and parts of the skin. Humans and normal flora have formed a symbiotic relationship over a period of time. With numerous disease processes, the interaction between the host and normal flora can be interrupted. Unlike normal wound healing, which is complex and crucial to sustaining the skin’s physical barrier, chronic wounds, especially in diabetes, are wounds that fail to heal in a timely manner. The conditions become favorable for microbes to colonize and establish infections within the skin. These include secretions of various kinds of molecules, substances or even trigger the immune system to attack other cells required for wound healing. Additionally, the healing process can be slowed down by prolonging the inflammatory phase and delaying the wound repair process, which causes further destruction to the tissue. Antibiotics and wound dressings become the targeted therapy to treat chronic wounds. Though healing rates are improved, prolonged usage of these treatments could become ineffective or microbes may become resistant to the treatments. Considering all these factors, more studies are needed to comprehensively elucidate the role of human skin normal flora at the cellular and molecular level in a chronic injury. This article will review wound healing physiology and discuss the role of normal flora in the skin and chronic wounds.

## 1. Introduction

Skin wound healing is a highly complex and dynamic mechanism involving various regulatory cells and molecules integrating to complete the wound re-epithelialization cascade [1]. Once the cutaneous layer is broken, the embedded cellular and molecular substances within the skin layers will synchronize at the designated phases to initiate the healing mechanism. Even though cutaneous wound healing is a systematic process, the phases are overlapping; therefore, it is known as one of the most complicated biological processes in the human body [2]. A chronic wound can be described as a stalled wound or wound that cannot heal in the expected time frame (of less than 3 months) [3]. It is characterized by an abnormal phenotype of epidermis and dermis cells that originates from the wound bed. A chronic wound is caused by a local factor (infection), systemic factor (diabetes), or both. The local factor affects the wound healing recovery of a particular wounded area of the skin, while systemic factors refer to the progression of the wound healing altered by the medical condition or history [4]. Generally, the chronic wound is a healthcare and socioeconomic burden. Approximately 2% of the population in developed countries has the potential to develop chronic wounds, especially leg ulcers, once in a lifetime [5]. Chronic wounds affected around 5.7 million people in the USA alone, at the cost of $20 billion for treatment and management yearly. The incidence is predicted to rise significantly in the elderly due to diabetes [6]. Besides skin lesion and diabetes, there are various underlying diseases such as sickle cell anemia, calciphylaxis, systemic lupus erythematosus (SLE), skin disease, or impaired physiological states that include paralysis, malnourishment (lack of nutrients), aging, and poor mobility that can affect the sequence of healing events, resulting in non-healing or chronic wounds [7].

In diabetes, for instance, several complications can lead to chronic wounds that are microvascular or macrovascular. However, diabetic foot ulcer (DFU) is considered more alarming than any other complications as it has become the primary cause of morbidity and increased hospital care for diabetic patients [8]. In addition, poor vascular flow and life-threatening infections are the major causative factors in diabetic chronic wounds impairing the wound healing process [9]. According to Armstrong and his team (2017), the cost of treatment for diabetic foot care has exceeded the cost for common cancers, as the diabetic wound is responsible for more admissions than any other diabetic complication. The researchers further stated that diabetic patients who developed foot ulcerations are at two times higher risk of death within 5 years upon diagnosis than patients who are not diagnosed with foot ulcers [10]. Based on the 2015 prevalence data, the International Diabetes Federation (IDF) reported that approximately 463 million adults are living with diabetes in 2019 [11], and diabetic foot with lower extremity complications affects about 40 to 60 million people globally (International Diabetes Federation-Complications 2020). In addition, the World Health Organization (WHO) has estimated that diabetes will be the seventh foremost death cause in 2030 [12]. Recently, it has been reported that every 30 s, one leg is being amputated due to DFU in some part of the world [13]. For the past 10 years in Malaysia, there has been an increase in diabetic cases, mainly affecting individuals of 30 years or older [14] in which 15% of the diabetic patients developed DFU [15]. Meanwhile, a statistic showed that 17% of patients admitted to the General Hospital of Kuala Lumpur are mainly due to DFU [16].

## 2. Anatomy and Physiology of Skin

**Figure 1:**
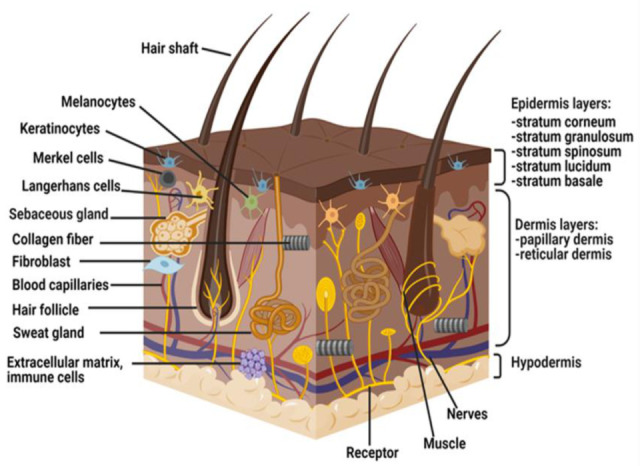
The anatomical structure of human skin.

The skin is the largest organ of the body and is divided into three layers; epidermis, dermis, and hypodermis, also commonly known as subcutaneous tissue [17] (Figure 1). The function of the epidermis is to protect the skin from microbial infections, chemical hazards, and mechanical as well as thermal hazards [18]. The epidermis also helps to maintain body temperature and prevent water loss through homeostasis [19]. 

The epidermis layer is composed of five stratum layers, keratinocytes, melanocytes, Langerhans cells, and Merkel cells. Beneath the epidermal layer lies the dermis or dermal layer that is rich with blood supply, extracellular matrix (ECM), nutrients, mast cells, nerve endings, lymphatic, fibroblasts, and epidermal appendages. The dermis provides structural integrity for the skin and modulates all cellular activities, including thermoregulation and cell restoration. The hypodermis acts as an insulator by trapping heat, providing shock absorption, and structural support to the skin [20,21].

## 3. Skin Wound Healing

Normal skin wounds take around one to two months to heal [22]. It is a natural, biological, and sophisticated process that occurs after an injury in tissue, which involves blood cells, connective tissue, parenchymal cells, ECM, and soluble mediators such as cytokines and growth factors interacting with each other during the wound healing mechanism. Classically, there are four major phases in wound healing; hemostasis, inflammation, tissue proliferation, and tissue maturation or remodeling [23,24]. The underlying connective tissue is exposed upon injury to the skin, and the collagens attract platelets to the injury site. This triggers platelet aggregation to deliver clotting factors such as prothrombin and fibrinogen, which initiates platelet clotting, coagulation, and a complement cascade through extrinsic and intrinsic pathways. During the coagulation phase, the platelets release chemicals from their granules into the plasma, including cytokines, growth factors, and pro-inflammatory mediators such as ADP, serotonin, Von Willebrand factor, and prostaglandin. These further assist the platelets in adhering to the injury site, forming a platelet plug, activating clotting chemicals, and maintaining vasoconstriction. Thrombin is a coagulation factor II that converts fibrinogen into fibrin, an insoluble protein, as a final product at the end of the complement cascade, thus arresting the blood flow at the wounded site. After clot formation, the coagulation process is switched off to prevent thrombosis. Fibrinolysis removes the fibrin and maintains vascular patency in balance with blood coagulation and fibrin formation [25,26,27,28].

In the inflammation phase, aggregated platelets secrete chemoattractant to activate the inflamed cells. These aggregated platelets release growth factors and pro-inflammatory chemokines to recruit neutrophils and macrophages to phagocyte the debris and fight against infection. Meanwhile, the endothelial cells from the wounded site are being stimulated to produce growth factors such as epidermal growth factor (EGF), transforming growth factor-beta (TGF-β), and platelet-derived growth factor (PDGF) to synthesize fibroblasts. After neutrophils have cleared out debris from the injury site, they undergo cell death or return back into circulation. Macrophages predominate the inflammation phase by clearing the apoptotic neutrophils, unwanted necrotic tissue, dead cells, and toxic production from the site. Macrophages transform into M1 (pro-inflammatory) and M2 (anti-inflammatory); M1 macrophages modulate cytokines such as interleukin (IL)-1β, TNFα, IL-6, IL-12, and matrix metalloproteinase (MMPs) while M2 macrophages produce arginase, TGF-β, CCL18, PGE2, and IL-10. These macrophages have their distinct lineages respective to their specific functions. For example, M1 macrophages are responsible for wound clearance of microorganisms and inflammation effects on the wounded bed, while M2 macrophages stimulate an anti-inflammatory effect, modulation, and encourage wound adhesion. On top of that, M2 macrophages also help in reducing inflammation and promoting angiogenesis and tissue regeneration in the healing process [29,30]. This is followed by the proliferation phase in which fibroblasts, keratinocytes, and endothelial cells migrate, proliferate, and re-epithelialize through the denuded wound to form new blood vessels [31,32].

During this time, new ECM are produced and some of its constituents such as collagen, elastin, proteoglycans, and hyaluronic acid aid in the construction of the granulation layer to restore platelet clot formation and contracting the wound size [33,34]. In a normal wound, keratinocytes relocate to the wounded area as a cell sheet over the granulation tissue and differentiate to re-epithelize the skin via integrin-mediated binding interactions with ECM molecules. However, these cells become dysfunctional and fail to regenerate the damaged tissue in the chronic wound [35]. While it undergoes a remodeling or maturation phase, the wound typically swells, reddens, and becomes itchy. The newly formed granulation tissue contains collagen fibers, becomes more vascular, and decreases the number of cells or changes in cell size. Additionally, the epidermal layer is also constructed, whereby this layer is subjacent to the basal lamina through the external and internal layers of the wound. At the same time, all unused proteins are degraded [36]. The collagen is remodeled from collagen type III to collagen type I, regulated by MMPs, while myofibroblasts produce collagen fibers for wound contraction [37]. In addition, myofibroblast highly expresses cytoskeleton α-actin, a type of smooth muscle protein that is essential for the contraction of the blood vessel [38]. At the final stage of wound healing, the myofibroblasts will be destroyed while cross-linked collagen fibers are rearranged and regulated by growth factors to give the newly formed scar a better tensile strength [39]. Table 1 shows the difference between acute and chronic wounds.

## 4. Pathophysiology of Chronic Wounds and Diabetic Foot Ulcers

**Figure 2:**
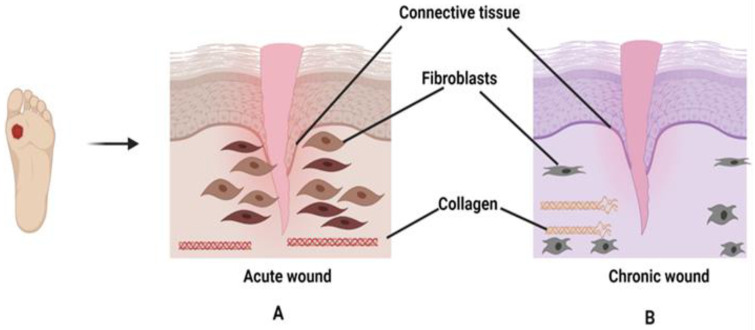
Diagram (**A**) shows the normal morphological structure of fibroblasts in an acute wound. In Diagram (**B**), the fibroblasts become senescent, reduce in numbers, inhibit migration to the wounded site, and alter collagen synthesis.

Healthy skin is essential for wound recovery when a minor injury is inflicted on the skin layer. This could be only possible with the association of molecules and cells residing within the skin. In chronic wounds, the cells are unhealthy or in abnormal conditions that cause prolong wound healing or showed no improvement. Generally, fibroblasts play a critical role in wound healing by synthesizing ECM (fibronectins, hyaluronan, and proteoglycans) and collagen [44]. Unlike acute wounds, fibroblasts in chronic wounds become senescent, decrease in proliferation, and migration to the wounded area is inhibited thus, hampering collagen production and deposition [45], as shown in Figure 2. Consequently, fibroblasts are unable to remodel the ECM, causing elevations of enzymes such as MMPs, collagenase, serine protease, and elastase. MMP is a type of zinc endopeptidase that can deteriorate the ECM components. There are 23 types of MMP classifications, however, only collagenase (MMP-1, MMP-8) and gelatinase (MMP-2 and MMP-9) actively participate in the wound healing stages. Under normal conditions, the secretions ratio between MMPs and tissue inhibitors of metalloproteinases (TIMPs) are equal. However, the imbalance expressions of MMPs and TIMPs can cause degradation and inhibition of growth factors and ECM components thus, delaying the wound healing process [46,47,48]. Keratinocytes are the building blocks for the epidermis, which trigger the release of cytokines when in contact with an injury or wound [49]. They also involve in the initiation, maintenance, and completion of the wound healing process to restore the epidermal barrier [50]. Keratinocytes at the wound edge become stalled in proliferation or hyperproliferative and show poor migratory activity to re-epithelialize, suggesting no improvement in wound recovery. However, the malfunction of keratinocytes during wound restoration in the molecular aspects is not clearly defined [51]. For example, the role of transforming growth factors (TGF-β) in wound healing is still elusive from the molecular aspects due to the paradoxical roles during the inflammatory phase. TGF-β either supports persistent inflammation by activating several pro-inflammatory cytokines (IL-1β, Tumour Necrosis Factor Alpha (TNF-α), and IL-6) or inhibits the activation of T cells to become effector T cells and encourage wound regeneration. In chronic wounds, some studies reported that high levels of TGF-β1 and β3 were observed in chronic ulcers, especially in the hyperkeratotic epidermis, while some studies observed low secretion levels of TGF-β in chronic wounds, which could be a possible reason for failure in wound closure. Overall, more evidence and in-depth understanding are needed to rule out the exact role of TGF-β in chronic wounds [51]. Cytokines such as TNF-α exhibit a negative effect on the healing process [52]. The increased production of TNF-α signifies prolonged inflammation, and once the dermis fibroblasts are destroyed, collagen and ECM production will stop [53].

**Figure 3:**
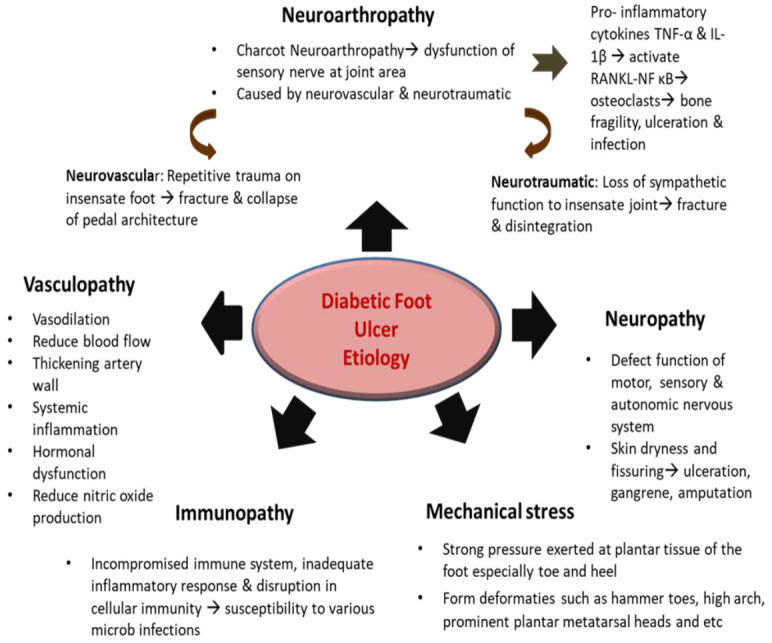
The pathophysiology of diabetic foot ulcer (DFU).

Diabetic skin ulcers present as a painful sore with the disintegration of skin layers including the subcutaneous tissue. The ulcerations are usually found on the lower limbs, especially on the foot, and are known as DFU [54]. In most cases, DFU causes severe destructions to the joints, bones, and soft tissues of the ankle and foot [55]. DFU can be categorized based on the depth of the wound, ranking from 1–5, starting with superficial ulcer right up to foot gangrene following the Wagner grade system [56]. In most DFU cases, chronic wound healing impairment leads to bacterial infection that can result in tissue destruction and, if untreated, could cause lower limb amputation [57]. Similar to normal wounds, ulcerated skin appears red and swollen, except that it is warm, and produces pus or excessive exudate followed by an unpleasant odor [58]. Additionally, the ulcerated foot can be seen with systemic infection, extensive cellulitis (more than 2 cm distant from the ulceration), bone necrosis, or gangrene, with reduced oxygen to the limb [59]. Apart from this, high glucose content in the blood produces substances known as advanced glycation end products (AGEs), which trigger the release of TNF-α and IL-1β that interrupt the synthesis of collagen and diminishes proliferation to re-epithelialize the wound [60]. High sugar levels also cause factors like thromboxane, Von Willebrand factor, factor VIII fibrinogen, and plasminogen activator inhibitor levels to be elevated in DFU patients. These factors not only contribute to poor platelet adhesion and thrombosis but also impede fibrinolysis [61]. The pathophysiology of DFU is multifactorial and the ulcerations can be generated not only by one but several factors, which include immunopathy, neuropathy, neuroarthropathy, vasculopathy, and mechanical stress [62]. Figure 3 summarises the pathophysiology of DFU.

## 5. The Role of Normal Flora as a Protective Agent towards Skin

The skin’s normal flora plays a vital role in the maturation and homeostasis of cutaneous immunity. The human skin harbors around 1000 types of bacterial normal flora. Theoretically, normal flora is referred to as the bacterial normal flora since bacteria typically populates the human skin without causing any harm to healthy individuals [63]. Most of these bacteria live in the superficial layers of the stratum corneum and the upper parts of the hair follicles [64]. Some bacteria, however, reside in the deeper areas of the hair follicles that are beyond the reach of ordinary disinfection procedures. These bacteria are the reservoir for re-colonization after the removal of the surface bacteria [65]. Other common types of bacterial normal flora that can be found on the skin are *Actino-bacteria*, *Firmicutes*, *Proteobacteria*, and *Bacteroidetes*. However, the most abundant type of bacteria that take up the huge space of the skin are *Staphylococcus epidermidis* [66] and *Staphylococcus aureus* [67] followed by *Staphylococcus haemolyticus*, *Staphylococcus hominis* [68], and *Micrococci* species [69].

A varying range of microbial flora inhabits the skin immediately after a person is born. Most of them are either mutualistic or show commensalism, and once they have established settlement, the skin microbial communities remain constant and stable on the skin over time [70]. The interactions between these microbe—host can be interrupted if some factors cause alterations to the skin structure, whether internally or externally [71]. These factors could include gender, age, medications (antibiotics), disease, different geographical regions, and lifestyle [72]. Haro et al.’s (2016) study on age and gender factors postulated that the microbes could change into different cell morphologies on the skin at the early and late years of a person’s life [73]. In addition, the difference of skin surfaces based on gender due to inconsistent secretions of hormones, skin pH, oil, and sweat could lead to favouritism for some microbes to populate the skin more than the others [74]. In relation to females, males have a greater abundance of lipophilic bacteria, especially Propionibacterium and Corynebacterium, because their skins are highly acidic, the enormous micro-colony size of bacteria, high production of sweat, and inadequate hygiene [72]. However, Shami et al. (2019) argued that gender did not influence the number or diversity of microbes on the cutaneous layer [69]. In addition, antibiotics are antimicrobial substances to inhibit proliferation or destroy microorganisms. In some skin conditions, these antibiotics profoundly obstruct the growth of normal flora and provide entry to opportunistic pathogens for disease exacerbation [75]. For example, patients with rosacea indicated that *Cutibacterium acne* protected the skin against harmful microbes by converting the oil into fatty acid, which could acidify the skin surface and inhibit colonization of pathogens [76,77]. However, in a different case with acne, antibiotic minocycline suppresses *Cutibacterium acne* thus, increasing the growth of *Streptococcus* species and *Pseudomonas* species while destroying other microbial flora that inhabit the skin [78]. A study was conducted to observe the behavior of skin normal flora among children and teenagers in rural and urban areas. The findings revealed that the type of outdoor activities and some physiological changes that occur due to different age range could diversify the dynamics of the normal flora on the skin [79]. Lifestyle, particularly hygiene and cosmetics, also influence the dynamics of the normal flora [80]. Even though soaps, cosmetics, and other body shower products help clean the skin, they can eradicate the healthy normal flora already residing on the skin, which could lead to the growth of foreign microbes [81].

The bacterial survival and the extent of colonization partially depends on (1) exposure of the skin to a particular environment, (2) innate and species-specific bactericidal activity on the skin, and (3) a high degree of specificity involved in the adherence of bacteria to the skin epithelial surfaces. Most bacteria do not attach to the skin. *Staphylococci* are mostly found as nasal flora, and this species outnumbered the *viridan streptococci* in conquering the nasal mucosa site. On the contrary, *viridan streptococci* can be seen dominating the oral canal but not the skin or nose [82,83]. The colonization of normal flora depends on the part of the body that is suitable for their growth, which includes oily (face), moist (armpit), or some dry environments (forearm and buttock). *Staphylococcus* species and *Propionibacterium* are commonly found in the oily part of the body, while *Corynebacteria* and *b-Proteobacteria* inhabit the moist areas of the body. The dry environments are occupied mainly by bacteria such as *b-Proteobacteria*, *Corynebacteria*, and *Flavobacteriales* [84,85]. The skin normal flora harmonizes with various innate factors, including complement, IL-1α, and antimicrobial peptides (AMPs) [86]. Normal flora release substances called phenol soluble modulins (PSMs) and bacteriocins, particularly from *S. epidermidis* and acnecin from *P. acnes* (or also known as *Cutibacterium acne*), to maintain the skin barrier from harmful microbes [87]. On top of that, *S. epidermidis* synthesizes secretome to reduce skin inflammation caused by *S. aureus* during an allergic reaction. This will be achieved by omitting several factors such as peptidoglycan, lipopeptide LP01, lipoteichoic acid (LTA) acid, and activation of IL-10 through modulation with innate and adaptive immunity [88]. *S. epidermidis* also induces the secretion of AMP (human beta defensin, HBd-2 and cathelicidins, and LL-37) produced by epidermal keratinocytes and skin commensal organisms. Then, it activates the toll-like receptor (TLR)-2 signaling pathway through the innate immune system. This response is important to promote the eradication of pathogenic bacteria and encourage wound healing [89,90].

*S. epidermidis* is able to increase the frequency of CD8 T cells along with IL-17A or IFN-γ to enhance the epidermal barrier and limit pathogen invasions [91]. Besides this, a reduced number of skin resident microbes with an increase of *Staphylococcus* species and *Corynebacterium bovis* were detected in a mice model of atopic dermatitis (AD). The findings showed that lack of skin normal flora, dysbiosis, and overgrowth of both *S. aureus* and *C. bovis* are responsible for acute atopic flares in patients diagnosed with AD [92]. Despite this, there is another mechanism utilized by skin normal flora, known as pattern recognition receptors (PRRs), which secrete nucleotide-binding oligomerization domain containing 2 (NOD2) that bind to peptidoglycans of Gram-positive and Gram-negative bacteria [93]. The mechanism is equally important because it helps the skin commensals recognize potential pathogens and initiate the innate immune system for further elimination. NOD2 with TLR2 and TLR6 are more specific to protect the skin from *S. aureus*, and likewise, TLR 2, 3, 7, 8, and 9 act as a defender against infections with herpesviruses, papillomaviruses, and poxviruses [94].

Besides bacteria, other types of microbes reside on the skin, such as fungi, viruses, and parasites considered normal flora of a healthy skin [95]. Examination of 14 different body locations such as foot, heel, toenail, and toe web of 10 healthy adult participants showed that *Malassezia* fungi accountable for most fungi colonization on healthy skin [96]. These fungi species are found in large numbers on oily skin containing lipid secreted by the sebaceous gland [97]. Although the benefits of *Malassezia* species to the skin host are still unclear, a study showed that this fungi species might enhance the activity of the epithelial cells by synthesizing aryl hydrocarbon receptor (Ahr) ligands that act as a shield against UV light [98]. *Aspergillus* and *Penicillium* are also commensal fungi, however, they are expressed in a lower numbers. Meanwhile, *Candida* and *Dermatophytes* can be either commensal or parasitic depending on the skin condition of the host [99]. For example, *Candida albicans* increases the activation of antigen-specific Th17 cells and destroys any non-commensal fungi that inhabit the local tissue by binding to their respective epitopes [100]. The bacterial–fungi interactions possess more virulence and resistance properties than with a single organism. The biofilms of both, containing *Staphylococcus epidermidis* and *Candida albicans*, exhibit resistance towards antibiotics compared to their single biofilms [101]. An in vivo study on mice was conducted to investigate the effects of the papilloma virus on carcinogenic skin cancer by introducing the strains into the immunocompetent mice. The investigation revealed that the commensal papilloma virus could trigger T cells to reduce the virulence of the virus, hence suppressing the growth of cancerous cells in the immunocompetent mice [102,103,104].

## 6. The Role of Microbes in Wound Healing

### 6.1. Acute Wound Healing

Normal skin aims to maintain its integrity by controlling the skin inhabitants from becoming harmful to the skin or preventing any penetration into the underlying tissue [105]. A slight alteration on the skin barrier may cause clinical changes to the skin, such as skin inflammation, allergic reaction, skin infections, formation of tumors in the superficial layer of the skin, and impaired skin healing [106]. Like other living organisms, microorganisms need essential requirements such as suitable temperature, pH, and nutrients for them to survive and multiply [107]. This can be achieved through the open cutaneous wound, however, the total population and the type of microorganisms depend on the severity of the wound and the immune level of the host [108]. The normal pH of healthy skin for both males and females is between 4 to 5, which is acidic to significant fractions of the human body [109]. The acidic condition is one of the ways to protect the skin from exogenous microorganisms [110]. The acidic pH of the skin is a desirable condition for *S. epidermidis* to preserve and attach to the skin, whereby this condition is unfavorable or could inhibit the growth of some skin pathogens such as *S. aureus* and *P. acne*. Most skin pathogens prefer an alkaline environment [111]. The pH of the wound either facilitates the growth or suppresses the colonization of pathogenic bacteria into the skin [112]. Under acute wound healing, when the wound becomes acidic, the amount of lactic acid and oxygen will reduce the pH of the skin. Acidic condition of the wound is needed for macrophages response, multiplication of fibroblasts, angiogenesis, collagen synthesis, and DNA formation to facilitate wound closure. In contrast, a wound that develops infection appears to increase in an alkalinity environment [113,114]. Temperature influences the wound healing rate. Any temperature that falls below the average body temperature will delay the wound healing process. In normal wound healing, the temperature will be elevated in the first few days before returning to the normal temperature due to the activities of the immune system and other physiological responses related to the healing process [115,116].

Recently, a study showed that exposure to UV radiation elicited an immune response through the synthesis of AMPs; however, concurrently, it caused immune suppression of specific cytokines and other reactions such as skin cancer, photoallergic, and phototoxic. Therefore, normal flora plays an important role in protecting the skin caused by ultra-radiation, thus preventing immune suppression [117]. Besides, skin normal flora can also elicit some other diseases such as impetigo, cellulitis, and systemic diseases, including endocarditis and sepsis [118]. When bacteria propagate inside the wound, they produce small chemicals used as signals to communicate with each other, as well as affecting the host’s immune cells and blood vessels. Upon detection, immune cells respond to these signals to kill the bacteria and limit the spread of the infections. One example is when epidermal keratinocytes contact pathogenic microbes, they will release a range of cytokines and chemokines to initiate a defensive response. This, in turn, will activate the production of human beta-defensin, cathelicidin, RNase-family antimicrobial peptides, and reactive oxygen species to protect the skin layer from further invasion by these pathogenic microbes [118]. A study conducted by Yang et al. (2017) postulated that re-epithelialization of epithelial cells is important for a wound to recover due to the capability of the cells to proliferate, differentiate, and protect from microbe infections. This can be achieved through IL-27, which is known to have a functional role in regulating wound re-epithelialization. The study also hypothesized that IL-27 secreted by dendritic cells and macrophages exhibited antiviral response through JAK/STAT3 signaling pathway. In addition, the expression of IL-27 is elevated in an in vitro model of mice skin after wound closure [119].

**Figure 4:**
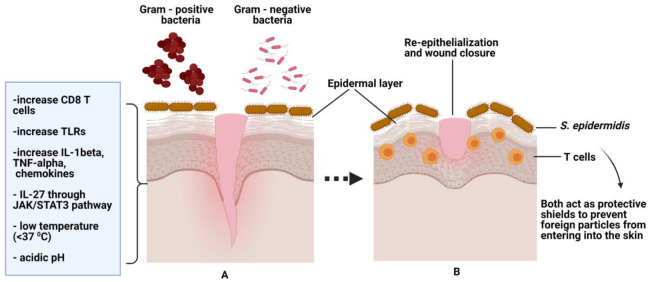
(**A**) shows normal flora/skin microbiome *(S. epidermidis)* on normal wound healing. The skin is protected from gram-positive /negative bacteria by *S. epidermidis*. The left corner shows factors that contribute to wound healing and further preventing colonization by skin pathogens. (**B**) shows both *S. epidermidis* and T cells help in wound closure and act as protective shields against pathogen invasions.

Recent studies also found that *S. epidermidis* plays an active role in skin immunity by activating cytotoxic CD8 T cells (CTLs) that belong to major histocompatibility complex Ib (MHC1b), and conferred N-formyl methione peptides on antigen-presenting molecules with the help of dendritic cells to CTLs for further actions. The studies also included that RNA gene sequencing on CD8 T lymphocytes was stimulated by *S. epidermidis*. The analysis results showed that immune regulation and genes associated with tissue repair were up-regulated. Through this pathway, both the bacteria and T cells prevent any foreign particle invasion and help hasten skin wound healing [106,120]. Moreover, a similar finding has been proven in skin biopsy samples whereby induced wound injury exposed to *S. epidermidis* drastically improved re-epithelialization and tissue granulation [106]. When an infection occurs, macrophages at the wounded site get in contact with pathogens and secrete cytokines such as TNF-α and IL-1β to regulate the endothelial cells to synthesis leucocytes’ adhesion molecules and chemokines. These will regulate the activity of leucocytes and help them to relocate to the infected area. Basically, leucocytes only move to the injury site after macrophages detect pathogen-associated molecular patterns (PAMPs) of the microorganisms through toll-like receptors and activation of the immune system [121]. An adopter molecule in the TLR signaling pathway known as MyD88 also accelerates the wound healing process since the MyD88 pathway assists in the production of granulation tissue, wound closure, and angiogenesis [122], as shown in Figure 4.

**Figure 5:**
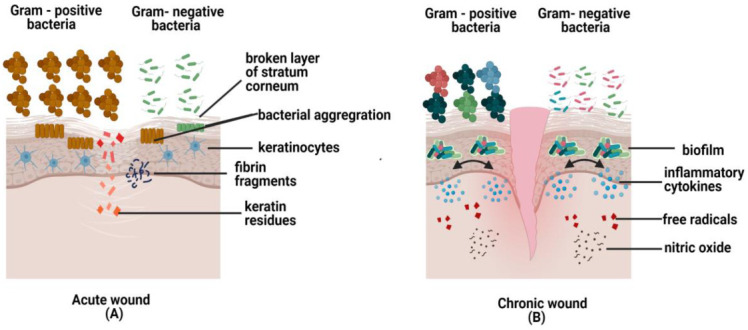
The figures above show microbes in acute and chronic wounds. (**A**) In acute wounds, fibrin fragments and keratin residues are favorable conditions for pathogens invasion into the skin. Hence, bacteria aggregate to prevent immune cells from destroying the bacterial community; (**B**) In chronic wounds, the biofilm formation secretes inflammatory cytokines, free radicals, and nitric oxide, which are toxic to cells and cell apoptosis.

Bay et al. (2018) conducted a clinical study to observe bacterial infections that occurred in the wound at the superficial layer of the epidermis. The group concluded that bacteria are always looking for any opportunity to reproduce and multiply, especially when there is a region that contains all the necessary conditions for them to survive. Such a situation happens when there is an entry into the skin and favorable conditions for the bacteria to colonize and multiply excessively. Examples are keratin residues from the broken layer of stratum corneum and fibrin accumulation at the borderline of the wound bed. This finding assumes that bacterial aggregation happens because the immune cells cannot prevent bacterial entry or immune cells are not allowed to enter the particular region colonized by bacteria (Figure 5). They also concluded that bacterial accumulation is present in all types of wounds except in chronic cases, which is more notable [123]. During acute wound healing, fibroblasts synthesized keratinocytes growth factor I to produce more keratinocytes and move to the wounded site. A recent study used a porcine model to evaluate the effects of the uninfected and infected wound with single species, followed by the wound co-infected with both *P. aeruginosa* and methicillin-resistant *S. aureus* (MRSA). The findings demonstrated that epithelialization could not occur due to low regulation of keratinocyte growth factor 1, as shown by decreased synthesis of fibroblasts in the co-infected wound. This could explain that mixed-species directly influence fibroblasts’ production, thus impairing the wound healing process [124]. An investigation conducted on pro-inflammatory interleukin IL-17A by using knockout mice (KO) demonstrated that wounds gradually healed and control activity of neutrophils against skin pathogens was observed in normal wounds compared to the wild mice with recombinant IL-17A. However, Vγ4 T-derived IL-17A is the main compound that converts IL-17 to inflammatory cytokine, impeding the function of dendritic epidermal T cells (DETCs) from producing keratinocytes and halts the migration of damaged tissue. This indicates that IL-17A could be another cytokine responsible for poor wound healing if secreted excessively [125]. In most acute wound cases, Gram-positive bacteria outnumbered Gram-negative bacteria. This can be seen in acute wounds whereby *Staphylococcus* species populations are greater than *Pseudomonas* species alongside skin undergoing wound healing [126].

### 6.2. Chronic Wound Healing

There are few classifications of microorganisms that are accountable for infection in a chronic wound. Such microorganisms are Gram-positive bacteria, including *Staphylococcus aureus* and *Staphylococcus epidermidis*. The Gram-negative bacteria are *Escherichia coli*, *Proteus mirabilis*, *Pseudomonas aeruginosa*, *Enterobacter* species, and *Morganella* species. Compared to the rest of the microbes, *S. aureus* is mostly resistant to antibiotics and contains high virulence, contributing to the pathogenicity of the host [127]. Furthermore, the infectious bacteria present in the wound bed tend to degrade the ECM and growth factors. The bacteria that already conquered the wound sheet generally produce biofilms acting as a barrier to allow them to grow, multiply, and protect from immune cells or become resistant to the antibiotic. The biofilm architecture comprises a fraction of bacteria implanted in the extracellular polysaccharide matrix or extracellular polymeric substances (EPS). Additionally, this biofilm is toxic to the other skin cells, explaining the delay in wound healing [46,128,129]. The EPS is water-based, containing a matrix with some protein substances that help channel nutrients, movement, and communication between the bacterial communities in a biofilm. In brief, EPS is the main component of most bacterial biofilms that support colonization or recolonization, by adhering to the wounded surface area [125,130]. Most bacteria exhibit some type of chemotaxis for movement, colonization and cause disease in a host. Chemotaxis can be defined as receptor-mediated, directed cell movement in a soluble chemical attractant concentration gradient known as a chemoattractant. Skin pathogens, especially *S. aureus* and *S. pneumoniae*, are incapable of being chemoattractant use various adhesion methods to attach themselves onto the surface of host tissue or cells and utilize the available nutrients while dispersing the virulence contents into the host. The adhesions include pili, fimbriae, and lipoproteins [131].

Bacterial biofilm formation is one of the indicators for its presence in a chronic wound. More than 50% of the biofilms are detectable via microscopic observation compared to only 6% in an acute wound. The bacteria species and the relative number varied from one wound to another [132,133]. Some anaerobic bacteria can survive and multiply deeper in a biofilm even though the oxygen level is depleted. Therefore, it is vital to investigate the type of bacterial strains and their relationship within the wound since it is insufficient to kill the biofilms cells by looking into the bacterial colonies alone [133]. In another study, Iwase and colleagues (2010) showed that *S. epidermidis* can block biofilm formation by *S. aureus* [134]. Similarly, *S. epidermidis* can form biofilm in chronic wound infection through implanted devices and host tissue, which can lead to septicemia [135]. The biofilm of *S. epidermidis* contains a protein known as polysaccharide intercellular adhesion (PIA), encoded by the *ica* operon gene, which helps in the formation of biofilm and adhesion by the *Staphylococcus* species [136]. PIA or polymeric-N-acetyl-glucosamine (PNAG) is a complement activator produced by C3 and C5. On most occasions, *S. epidermidis* can produce PNAG-dependent biofilms as a shield to protect itself from IgG and C3 factor, inhibit phagocytosis and destruction by neutrophils and prevent the antibody from reaching the surface of the biofilm. Through this, neutrophils’ reactions could be diverted and become ineffective in destroying *Staphylococcus* species [137]. Unlike methicillin-resistant *S. aureus* commonly found in other anaerobic bacterial colonies or mainly joined with Gram-positive bacteria, *Pseudomonas aeruginosa* independently (planktonic state) can produce biofilm resembling archetypal mushroom with enclosed ECM [138].

Several pathways are involved in the production of *Pseudomonas aeruginosa* biofilm. One of the pathways is cyclic diguanosine-5′-monophosphate (c-di-GMP), which will enhance bacterial cell attachment, cell clumping, and EPS release to the surrounding wound that causes severe infections [139]. Complement cascade activation in the skin wound has shown some conversions in wound healing [140]. It is considered that complement activation helps protect against infection and inhibits wound healing if the activation is dysregulated, especially CD59. Dysregulation of CD59 increases cytokine release, cell multiplication, and inflammation that can cause damage to the tissue [141]. During *Staphylococcus* species invasion, the complement cascade is stimulated by three separate routes that activate the C5a and C5b factors. C5a captivates neutrophils for bacterial engulfment, whereas C5b produces a toxic vent to target the bacterial membrane and destroy gram-negative bacteria [142]. On the contrary, Gram-positive bacteria are impenetrable by membrane attack complex due to their thick peptidoglycan membrane [143]. PTX3 is a substance produced by antibody-mediated immunity that works with plasminogen and fibrin in the coagulation phase. In a chronic wound, PTX3 acts as a mediator between antibody-mediated immunity and cellular pathways to activate phagocytosis when in contact with any infectious microbes [144]. In a chronic wound, due to some catabolic reactions, the oxygen level will reduce the skin′s pH, hence altering the wound healing phases such as collagen production, blood vessel formation, and immune system [113]. Chronic wounds are characterized by high numbers of Langerhans cells, neutrophils, pro-inflammatory macrophages [42], pro-inflammatory cytokines, reactive oxygen species [145], and protease [146]. Similar to normal wound healing, these immune cells received signals from innate cellular immunity and propagated the signal to the infected wound bed for further actions. However, the immune cells are unable to perform their regular duty as they become defective to function. The dysregulation of neutrophils and macrophages becomes less effective in inflammatory effect and phagocyte the bacteria, contributing to the delay in wound healing [27,42]. Neutrophils utilize neutrophil extracellular traps (NETs) to kill infectious microbes or biofilms by releasing chromatins and granular protein contents. It is also known as NETosis. NETs accomplished their tasks using two strategies. The first is to destroy or stop the proliferation of pathogens, and the second is to disable the migration of the pathogens. Despite its role against microbe infection, accumulated evidence has shown that microbes can surpass NETs antibacterial activity and become resistant to it by degrading NETs by enzyme nucleases and inhibiting NETs synthesis [29]. In addition, *S. aureus* is able to induce extracellular trap formation by releasing leucocidin from the biofilm to escape the antimicrobial activity of NETs. This encourages the multiplication of bacterial colonies to disperse to a new place for new biofilm formation; thus, helps the bacteria to sustain and survive in the chronic wound for a longer time [147]. As the focus has been mainly on the diverse bacterial pathogens in chronic wounds, the fungi’s role in the wound is considered significant. The imbalance number of commensals and pathogenic fungi residing at the skin barrier also contributes to the delay of wound healing. *Candida* species and *Trichosporon* species could hinder wound healing either via its spores or biofilms disposition to exacerbate the inflammation and consume oxygen contents resulting in necrosis [138,148]. A study has illustrated that *Candida albicans* could defect the role of macrophage and cell apoptosis in cutaneous wound healing [149]. IL-27 produced from CD301b+ immune cells could exhibit antiviral properties suggesting its vital role in skin regeneration [119,150]. It is thought that IL-27 could be able to quell the infection caused by the Zika virus through the activation of STAT 1 signal transducer and activators of transcription 1 (dependent) to STAT 2 (independent) [151]. A different virus has different ways to seize the host for replication. For example, in a cutaneous wound, Herpes simplex virus (HSV) replicates in the epidermis by forming glycoprotein, fused, and attaches to the keratinocytes before disseminating its viral particles to initiate infection. Many studies have investigated the skin normal flora or microbiome related to protection and thermoregulation, however, studies on the role of fungi and virus as cutaneous residents are still scarce [152].

The severity of DFU corresponds to the amount of *Staphylococcus* species present in the wound. The time taken for ulcer formation depends on the diversity and level of *Proteobacteria* in the wound [126]. Chronic ulceration of diabetes could be caused by more than one type of skin commensals, producing a synergetic effect that converts non-virulent bacterial species to virulent and causing damage to the host. This has been proved based on the high throughput of 16S rRNA gene sequencing [153]. Recently, endotoxin secretion from Gram-negative bacteria has been observed in diabetes patients and it is likely to induce insulin inefficiency through the elevation of pro-inflammatory cytokines in the adipocytes such as TNF-α [154]. During the pathogenesis of chronic wounds, an infection caused by *S. aureus* increases glucose resistance by blocking the insulin to its target site hence elevating the glucose level in the blood [155]. Besides that, biofilms’ continuous presence could delay wound healing in the DFU by releasing the inflammatory cytokines, free radicals, nitric oxide, and complement initiation through the activation of immune cells [156] as shown in Figure 5. In diabetic conditions, neutrophils become hyperactive and secrete high amounts of TNF-α, which will increase the process of NETs formation to destroy neutrophils, hence impairing wound healing [157]. In contrast, some skin bacteria, either Gram-positive or Gram-negative, can regulate the NETs formation by releasing the exotoxins to suppress the activity of skin pathogens, thereby boosting the defense system and mediating phagocytosis [29]. The chronic skins of diabetic patients have shown a higher number of mast cells and macrophages [158,159], while T cell receptors and CD4 T cell numbers are reduced [160]. The presence of these cells in chronic wounds could explain the prevention of wound recovery, promoting skin infection, and jeopardizing the wound healing system [159].

A study to examine the effects of the *Circoviridae* virus on two different groups of children with Type I diabetes and a control group revealed that *Circoviridae* species is more pervasive in the control group than in diabetic children. However, most children did not exhibit any signs and symptoms after being infected with this virus and showed no significant difference in both groups [161]. Although some studies reported that the virus could protect from developing type 1 diabetes, some studies revealed a contradictory report. It seems that virus infection in murine models induced with type I diabetes shows impairment in the function of pancreatic Langerhans β-cells and autoimmunity activation, which could eventually lead to cell destruction and apoptosis [162]. In diabetic wound healing, the increased level of TLR especially TLR2 signaling pathway proteins (MyD88, pIRAK, and TRIF) and their inflammatory cytokines (IL-1β and TNF-α) were high in induced mice compared to non-induced mice. The significant increase of these levels in diabetic mice did not show any improvement in wound healing, however, it extended the duration of the inflammation phase [122]. Germ-free mice (without commensals) showed increased wound epithelialization, wound closure, and angiogenesis with lesser scar formation than non-germ-free mice (with commensals). These mice showed persistent inflammation, cytokine release, and wound cessation, concluding that the presence of skin normal flora decreases the efficiency in wound healing [163]. On the contrary, mice that have been induced with different types of antibiotics before wound incision showed decelerated wound healing than mice without antibiotics [126]. 

## 7. Treatment for Chronic Wounds and Diabetic Foot Ulcers

The use of antibiotics in treating chronic wounds is challenging since the microorganisms, especially bacteria, tend to become resistant to the prolonged treatments with no improvement in wound healing besides causing kidney failure and oral tract infection. The antibiotic treatments can be administered to the patients either by parenteral, oral, or topical [164]. Since some antibiotics can reduce a type of pathogen while increasing the growth of other pathogens, an empirical antibiotic choice should be carefully selected based on the clinical examinations, the severity of the infection, antimicrobial sensitivity pattern, and the aetiological agent. A broad spectrum of parenteral antibiotics is administered for severe infections, while narrow-spectrum oral antibiotics are administered for mild infections [165].

Besides drugs, there are different types of wound dressings applied for wound treatments, including passive dressings (such as gauze), interactive dressings (such as foam or sponge), advanced dressings (such as alginate or hydrocolloid), bioactive dressings (such as Alloderm and Apligraf), and antimicrobial dressings (such as a topical antibiotic). The goal of a good dressing is to retain moisture for wound closure, prevent infections, reduce pain or irritation, and scar formation. Additionally, a selection of dressing also depends on the application and type of wound. For example, a gauze dressing is used in a shallow flat wound for less exudate production, while foam, sponge, and alginate are suitable for minor burn or deep wounds to absorb excessive exudate from the wound. Bioactive or tissue-engineered dressings accelerate wound healing by mimicking the natural function of ECM and mediating the physiology of healing phases through angiogenesis, cell proliferation, and new tissue formation. Antimicrobial dressings are mainly applied topically to chronic wounds that are infected with bacteria [6]. Traditional wound dressings are mainly used to maintain the dryness of the wounds and prevent infections but cannot absorb a large number of exudates. Different strains of normal flora and pathogens show different interactions and impacts on the usage of the wound dressing. Another study reported that besides improving the physicochemical properties of the dressing materials, loading additional substances into the dressing materials could also prevent microbial infections [166]. Jack and colleagues (2017) have used wood nanocellulose hydrogel suspension to observe the activity of *P. aeruginosa* from a wound. They found that reduced virulence factor and biofilm formation by *P. aeruginosa* is due to the material surface and porosity, which did not support the growth of this bacteria species. This study suggested that dressing made of wood nanocellulose could be a novel finding to prevent microbe’s growth and promote a moist environment for wound acceleration [166]. In a similar study, wood nanocellulose hydrogel crosslinked with ion (copper or calcium) showed inhibition growth of *S. epidermidis* and retarded *P.aeruginosa* biofilm formation [167]. In a different study, chitosan exhibited good antibacterial and antifungal effects towards bacteria (Gram-positive and Gram-negative) and fungi while maintaining its physical properties suggesting it is a good material for wound dressing [168]. Another study using thymol from natural monoterpenoid phenol integrated into wound dressing has demonstrated antibacterial properties and can destroy the biofilm formed by methicillin-sensitive *S. aureus* [169].

Total contact cast (TCC) is tagged as an alternative treatment approach for DFU. Although the healing rate significantly improved with TCC, yet countless side effects can be life-threatening, such as iatrogenic infections, ulcers, blisters, or skin abrasions [170]. Besides TCC, some other treatments for DFU are maggot debridement therapy (MDT), negative pressure wound therapy (NPWT), and dermaspace systems (DS) [171]. DFU patients who have severe wound infections or osteomyelitis need to undergo antibiotic therapy for at least a month without surgical intervention to resolve the infections as recommended by the Infectious Disease Society of America (IDSA) and the International Working Group on the Diabetic Foot (IWGDF) [58]. Wound dressings are also another choice of treatment for DFU. Some of the available dressings are hydrogel, foam, films, hydrofibers, hydrocolloids, acrylics, and calcium alginates. Cellulose/collagen-based dressing has shown some improvements in diabetic non-wound healing by accelerating the wound healing rate and improvement in wound closure [171]. In addition, the integration of antibacterial properties such as silver nanoparticles into the collagen wound dressing has the potency to reduce the risk of microbial invasions through the broken wound in DFU [172]. Generally, topical antibiotics are not the best choice to treat DFU due to the imbalance of moist production and contact dermatitis [173]. However, a recent finding proved that topical probiotics such as Kefir (cultured probiotic beverage) exhibit potent antimicrobial effects against *S. aureus* and *E.coli*. Probiotics help in reducing the possibility of skin infections and improve tissue regeneration by regulating skin microflora through various mechanisms in the skin [174]. Some other examples are tabulated in Table 2. The use of wound dressings alone does not help eliminate pathogens but, together with additional substances that exhibit some antibacterial/antimicrobial properties, could shed some light on treating chronic wounds, especially in DFU.

## 8. Conclusions and Future Perspectives

Collectively, the conclusions of these research papers has attributed to our knowledge of chronic wounds, the pathophysiology of wound healing, and interactions of normal flora-host depending on the multifaceted conditions of the host. Studies revealed that in healthy individuals, when cells are normal, the overall wound healing phases are well-orchestrated which last only a few days, but in chronic wounds, the majority of cells become debilitated, lost skin integrity, dysfunctional or adverse effects by immune cells to the skin and pathological changes that are difficult to be irreversible. Many underlying factors hamper the wound healing process; one is infection. Current technologies have portrayed a better characterization of normal flora and the potential of these commensals to become pathogen in any opportunistic niches in all stages of wound. The microbial adaptations to the microenvironment of the skin lead to virulence and impaired wound healing. Therefore, it is important to investigate further the role of microbes at the cellular and molecular levels and not only focus on bacteria but also other normal flora such as viruses and fungi to rectify new treatments for chronic wounds. The most challenging part of treating chronic wounds is polymicrobial infections and a high tendency to become resistant to prolonged antibiotic treatments. Loading additional antibacterial substances into the wound dressings, selecting suitable empirical antibiotics based on microbial profile and antimicrobial resistance pattern could reduce the infections and improve wound healing in chronic wounds. However, a deeper understanding of normal and pathological healing will illuminate the interaction between skin cells, normal flora, and their microenvironment. This information might help investigators develop better treatments or methods to eliminate the microbes present in chronic wounds completely.

## Figures and Tables

**Table 1:** Differences between acute and chronic wound healing.

Items	Acute Wound Healing	Chronic Wound Healing
Growth factors	Normal degradation [40]	High degradation [40]
Neutrophils	Normal activation of neutrophils to phagocyte the pathogens or foreign particles during inflammation [41]	High activation of neutrophils with excessive secretion of reactive oxygen species and ECM degradation [41]
Macrophage	During inflammation, ability to transform from pro-inflammatory macrophage, M1 to anti-inflammatory macrophage, M2 [30]	During inflammation, poor transformation of macrophage from M1 to M2 [30]
Anti-inflammatory cytokines	Decrease production [42]	Increase production [42]
MMPs and inflammatory cytokines	Decrease secretion of MMPs and inflammatory cytokines [42]	High secretion of MMPs and inflammatory cytokines [42]
Wound healing contaction/ contracture and Types of wound	Wound contraction Types of wound-normal scar wound [42]	Wound Contracture Types of wound; (a) chronic non-healing wound-ulcer, (b) dehiscence-scanty of wound healing, (c) fibrosis (hypertropic scars and keloids)—uncontrollable wound healing [42]
Pathological condition of epidermal skin	Epidermal skin still present as normal skin [27]	Hyperkeratotic (thick keratin layer or parakeraotic (anucleated keratinocytes) [27]
Duration of wound healing	3 months [43]	More than 3 months and up to 7 months (for active ulcer) [3]
Phases of wound	Normal four phases-hemostasis, inflammation, proliferation and remodeling [23]	Not normal phases—Prolonged inflammation, impaired proliferative and remodeling phase [23]
Cell mitosis	Takes place [41]	No cell mitosis [41]
Granulation tissue	Normal production [40]	Neoangiogeneis, less fibroblasts, low oxygen→ tissue hypoxia Tissue hypoxia—low production of granulation tissue [40]

**Table 2:** Types of antibiotic/antimicrobial therapy, route of therapy, targeted pathogens, and impact to wound.

Types of Antibiotic/ Antimicrobial Therapy	Methods of Application/ Route of Therapy	Targeted Pathogens	Impact of Therapy	References
Gentamicin-collagen sponge with systemic antibiotic therapy (levofloxacin with clindamycin or amoxicillin-clavulanate	Gentamicin collagen sponge–topical Antibiotic—oral or intravenous	*S. aureus, Streptococci, E. coli and P. aeruginosa*	Overall no significant improvement in healing No side effects caused by antibiotics	[175]
Cephalosporin agent (ceftaroline fosamil)	Intravenous	Gram-positive bacteria, MRSA	Effective in treatment Potential substitute for glycopeptide therapy	[58]
Amoxicillin—clavulonate or cefotaxime	Oral or parenteral	Anaerobic bacteria	Sensitive to the antibiotic treatment	[176]
Tobramycin	Oral or parenteral	Gram-negative bacilli	Effective in treatment	[177]
Linezolid	Oral or parenteral	*Staphylococcus* sp., methicillin-resistant staphylococcus (MRS), *Enterococcus* sp.	Effective in treatment	[177]
Pexiganan and nisin (dual Antimicrobial peptide-biogel)	Topical on collagen DFU 3-D model	*S.aureus* and *P.aeruginosa*	Eradication of *S.aureus* isolates in infected area Inhibitory activity of *P.aeruginosa* against the AMP	[178]
Collagen with gentimycin sulphate, doxycycline and vancomycine	Apply topically on patient’s infected wound	*Enterococcus* sp.	Wound healed with tissue formation and granulation	[179]
Metronidazole	Apply topically on patient’s infected wound	*S. aureus* and anaerobic bacteria	Wound healed and formation of scab over the large wound	[180]
Clindamycin	Intravenous	*S. aureus, S. pyrogenes,* polymicrobial (*P. aeruginosa, Klebsiella* and *Proteus)*	Effective in treatment (clearance of infection) from the wound	[181]

## Data Availability

Not applicable.
